# A Multisectoral Food-Assisted Maternal and Child Health and Nutrition Program Targeted to Women and Children in the First 1000 Days Increases Attainment of Language and Motor Milestones among Young Burundian Children

**DOI:** 10.1093/jn/nxz133

**Published:** 2019-07-03

**Authors:** Deanna K Olney, Jef L Leroy, Lilia Bliznashka, Marie T Ruel

**Affiliations:** Poverty, Health, and Nutrition Division, International Food Policy Research Institute, Washington, DC, USA

**Keywords:** children, language development, motor development, Africa, multisectoral program

## Abstract

**Background:**

Child development is affected by multiple factors throughout pregnancy and childhood. Multisectoral programs addressing these factors may improve children's development.

**Objective:**

We evaluated the impact of a food-assisted multisectoral nutrition program (*Tubaramure*) on children's (4–41.9 mo) motor and language development. *Tubaramure* was targeted to Burundian women and children in the first 1000 d and provided micronutrient-fortified food rations; nutrition, health, and hygiene behavior change communication; and health system–strengthening activities.

**Methods:**

Program impact was assessed using a cluster-randomized controlled trial with repeated cross-sections: 2010 (baseline, children 4–41.9 mo), 2012 (follow-up during implementation, children 4–23.9 mo), and 2014 (follow-up postimplementation, children 24–41.9 mo). Sixty villages were randomly assigned to 4 groups with varying timing and duration of food rations: pregnancy–24 mo; pregnancy–18 mo; 0–24 mo; and control, no direct *Tubaramure* benefits. Treatment groups were pooled and compared with control using difference-in-difference estimates. We examined impact pathways by assessing program impacts on intermediary variables and their associations with development outcomes.

**Results:**

At first follow-up, *Tubaramure* positively affected language (0.4 milestones, *P* < 0.05) but not motor development among children aged 4–23.9 mo. Among the 12–23.9 mo age subgroup, the program positively affected language (0.7 milestones, *P* < 0.01) and motor (0.6 milestones, *P* = 0.08) development. At second follow-up, among children aged 24–41.9 mo, *Tubaramure* marginally affected motor development (0.4 milestones, *P* = 0.09). In age subgroup analyses, program impacts were limited to children aged 24–29.9 mo [0.4 motor (*P* = 0.09) and 1.0 language (*P* < 0.01) milestones]. Pathway analyses revealed significant positive impacts on diet, health, and nutritional indicators of children aged 12–23.9 mo and health and nutritional indicators of children aged 24–29.9 mo, supporting the plausibility of program impacts on child development.

**Conclusions:**

*Tubaramure* had small positive impacts on children's motor and language development through multiple pathways, demonstrating the role multisectoral nutrition programs can play in improving children's development. This trial was registered at clinicaltrials.gov as NCT01072279.

## Introduction

Child development can be negatively affected by nutritional, health, and psychological insults during pregnancy. Postnatal factors including poor nutrition (caused by food insecurity or suboptimal feeding practices), health, and care can also negatively affect children's development (1–4). Exposure to multiple risk factors for longer durations has cumulative effects (2, 5–7). Interventions that reach children during the first 1000 d (and beyond) and address these multiple risk factors have the potential to improve children's developmental trajectories (8–10).

Multisectoral food-assisted maternal and child health and nutrition (FA-MCHN) programs targeted to women and children during the first 1000 d can improve many of the factors that affect children's development. These types of programs typically provide nutrient-rich foods to mothers and children and behavior change communication (BCC) designed to improve maternal knowledge and adoption of adequate infant and young child feeding (IYCF) and care practices; in addition, they work with the local health system to strengthen the provision of health services and encourage their use. Although the primary goal of these programs is to improve maternal and child nutritional status, this combination of interventions has the potential to address many of the factors that affect children's development such as household food security; maternal health, nutrition, stress, and caregiving practices; and children's diets, health and nutritional status.

Despite this potential of multisectoral FA-MCHN programs to improve children's development, we are not aware of any evaluations that have assessed their impact on children's language or motor development. However, improvements in children's development have been found in studies that have assessed the impact of some of the individual components of FA-MCHN programs. For example, improvements in motor development have been found in studies that provided nutrient-rich foods to undernourished pregnant women in Bangladesh (11) and fortified porridge to children 6–12 mo of age in South Africa (12). In addition, a study in Bangladesh demonstrated impacts of an intensive BCC strategy aimed at improving IYCF practices on language and motor development among children 6–47.9 mo of age (13). Furthermore, some of the expected impacts of multisectoral FA-MCHN programs targeted to children during the first 1000 d, such as improvements in children's nutritional status and reductions in morbidity, have also been linked to better developmental outcomes (1, 2).

In this article, we use data from the evaluation of US Agency for International Development-Food for Peace (USAID-FFP) multisectoral FA-MCHN program implemented in Burundi (*Tubaramure*, “Let's help them grow” in Kirundi) to estimate the secondary impacts of the program on children's motor and language development. The intervention had positive effects on reducing maternal and child anemia and child stunting (14, 15). This is the first cluster-randomized evaluation of a multisectoral FA-MCHN program that provides rigorous evidence of the impact of this type of program on children's motor and language development.

## Methods

### Program description

*Tubaramure* was a multisectoral FA-MCHN program implemented by a consortium of nongovernmental organizations (Caritas Burundi, Food for the Hungry, and International Medical Corps) led by Catholic Relief Services. The program was targeted to mothers and children during the first 1000 d. The program was implemented in the provinces of Cankuzo and Ruyigi in Eastern Burundi where the prevalence of stunting was among the highest in the country (16). *Tubaramure* provided a package of 3 primary intervention components: food [micronutrient-fortified corn–soy blend (CSB) and oil], health (health-strengthening activities and promotion of the use of preventive and curative health services), and care (health, hygiene, and nutrition BCC). Together, this package of interventions was expected to improve household food security; maternal diets, health, nutrition, well-being, and caregiving knowledge and practices; and children's diets, health, nutrition, and development. Additional information on the program design has been published elsewhere (14).

The food component of the program consisted of monthly distributions of family and individual rations of micronutrient-fortified CSB and oil from pregnancy through 24 mo of age (the timing and duration of the food component were varied for the evaluation). Pregnant women were eligible to enroll in the program from 4 mo of gestation. Details on the composition of the food rations and micronutrient composition of the CSB and oil have been previously published (14).

The health component focused on health-strengthening activities within the existing health system (e.g., training health staff and providing key supplies such as equipment for prenatal care, labor and delivery, growth monitoring, and curative care) (17). This component aimed to improve health services at local health centers. These services were available to everyone in the health centers’ catchment area (which could include collines from different treatment arms). The use of these strengthened health services was actively promoted through *Tubaramure*’s BCC activities and program beneficiaries were strongly encouraged to attend the requisite preventative care services and to seek curative care when necessary.

The BCC strategy, the central activity of the program's care component, used a cascade training system through which *Tubaramure* health promoters trained groups of leader mothers (program beneficiaries elected by their peers) who, in turn, each trained a group of beneficiary mothers. Training sessions at both levels were expected to occur twice a month and participation was encouraged throughout the program period. The curriculum for the BCC, developed by Food for the Hungry, consisted of 5 learning modules with 6–12 lessons within each module. The first module had 6 lessons related to the program's objectives, teaching techniques, the value of children, and ability to change, among others. The second and third modules, “Essential Nutrition, Hygiene, and Care Practices during Pregnancy,” and “Essential Nutrition, Hygiene, and Care Practices during Infancy,” had 9 and 12 lessons, respectively. These modules focused on nutrition, hygiene, and care during these 2 periods and included topics such as danger signs during pregnancy, maternal nutrition and micronutrients, malaria symptoms, prevention and treatment, pre- and postpartum care, early initiation of breastfeeding, exclusive breastfeeding, preventive health visits for infants and young children, child spacing, and point-of-use water treatment and safe water sources, among others. The fourth module largely focused on complementary feeding for children 6–23 mo of age and included a module related to preparing CSB with local foods to increase the diversity and nutrient density of complementary foods. The last module, “Management of Childhood Infections,” had 6 lessons focused on recognition, prevention, and treatment of childhood illness.

### Study design and participants

We evaluated the program's impact using a cluster-randomized controlled study (NCT01072279) with repeated cross-sections. Two hundred and ten collines (hill, an official administrative unit in Burundi) were selected for possible inclusion in the study if they fell between the first and 99th percentiles for colline population size and were not served by >1 health center each. These collines were divided into 5 strata (based on population size) in Cankuzo and 10 strata in Ruyigi and a random sample of 60 collines was drawn (4 collines from each stratum). These 60 collines were randomly assigned to 1 of 4 study groups (15 collines per group) that received monthly food rations for different durations: pregnancy to 24 mo (T24); pregnancy to 18 mo (T18); *Tubaramure* No Food during Pregnancy (TNFP): 0–24 mo; and control, no direct *Tubaramure* benefits (**Supplemental Figure 1**). All treatment groups (T24, T18, and TNFP), but not the control group, were encouraged to participate in BCC sessions and to attend preventative health visits. Attendance at BCC sessions and preventative health visits was not required to receive the food rations. The control group had access to the strengthened government health services but not to the BCC sessions or food rations.

Three rounds of data were collected between October and December, 2010 (baseline, children aged 0–41.9 mo), 2012 (first follow-up, children aged 0–23.9 mo), and 2014 (second follow-up, children aged 24–41.9 mo). The first follow-up was designed primarily to assess program impacts on IYCF practices and anemia. The second follow-up was designed primarily to assess program impact on stunting among children 24–41.9 mo of age who had the opportunity to benefit from full program exposure during the first 1000 d. In both rounds of data collection, we aimed to answer a secondary research question about the program's impact on child development outcomes. Within each study colline, we drew a random sample of households with children 0–41.9 mo of age at baseline (2010), 0–23.9 mo of age at the first follow-up (2012) and 24–41.9 mo of age at the second follow-up (2014). In households with >1 child in the eligible age range, children were listed in alphabetical order, according to their first name, and the first child on the list was selected as the “index child.” Additional information on the evaluation design has been previously published (14).

Information about the study was provided to prospective survey participants by trained fieldworkers. Written informed consent for participation was obtained from either the household head or the mother of the index child. The protocol was approved by the Institutional Review Board of the International Food Policy Research Institute and by the Ministry of Health of Burundi.

### Data collection and measures

#### Household surveys

We used household questionnaires to collect data on household demographic and socioeconomic characteristics, maternal characteristics, and child characteristics. Household food security was measured using the household hunger scale with a continuous score ranging from 0 to 6 (18). Maternal stress was assessed using the Self-Reporting Questionnaire (19). Because no research has been conducted to determine the appropriate cutoff for Burundi, we used a cutoff of 10 [i.e., a score >10 (out of 20) indicated a stressed mother] based on research in Rwanda, a neighboring country (20). IYCF practices were assessed by maternal recall and constructed as per the WHO indicators (21). We assessed child morbidity using maternal recall of illness for the past 2 wk. Children's motor and language development were assessed using parental report questionnaires. Mothers were asked whether their child could perform a set of 30 motor and 21 language milestones (**Supplemental Table 1**). These scales were adapted from ones previously used in Tanzania (22, 23). The items included in these scales were derived from other developmental measures including the Griffiths and McCarthy Scales (24, 25) and were ordered to reflect a generally accepted sequence of motor and language development. These scales were also used in Bangladesh for the evaluation of an intensive BCC program (13).

#### Clinical assessments

At each round of data collection, we also took anthropometric and hemoglobin (Hb) measures for mothers and children. For anthropometric assessments, trained staff measured children's length/height and mothers’ height using a wooden length/height board (Shorr Productions) and weight using an electronic scale. For children, length-for-age *z* scores (LAZs), height-for-age *z* scores (HAZs), and weight-for-height *z* scores (WHZs) were calculated using the 2006 WHO growth reference (26). To assess Hb, capillary blood from a finger prick sample was used immediately to measure Hb using the HemoCue system. Maternal anemia was defined as Hb <12.0 g/dL for nonpregnant women, women who did not know whether they were pregnant, or who were missing information on pregnancy, and Hb <11.0 g/dL for pregnant women. Hb values were adjusted for altitude (14). Maternal BMI was calculated (in kg/m^2^) and underweight defined as BMI <18.5.

### Statistical analysis

The analyses were restricted to children aged 4–23.9 mo for the comparisons between 2010 and 2012, and to children aged 24–41.9 mo for comparisons between 2010 and 2014. Within the 2 age groups, age subgroup analyses were also conducted to better capture age-dependent changes in child development.

**FIGURE 1:**
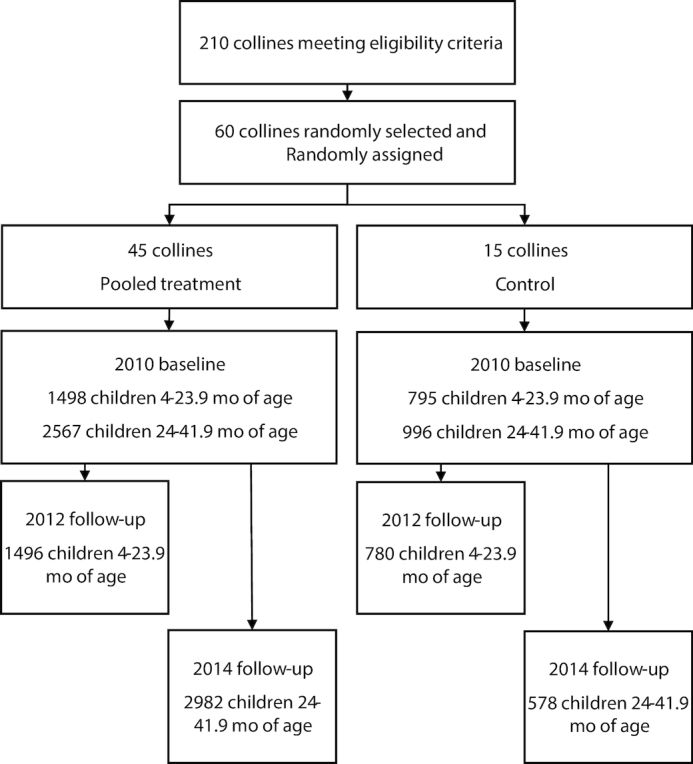
Sample sizes for the pooled treatment group and the control group in 2010, 2012, and 2014.

For the first comparison (2010 with 2012), the sample consisted of 2293 children at baseline (*n* = 1498 for pooled treatment and *n* = 795 for control) and 2276 children at follow-up in 2012 (*n* = 1496 for pooled treatment and *n* = 780 for control) (**Figure 1**). The analyses were conducted for the full sample and repeated for 4 age groups: *1*) children aged 4–11.9 mo (*n* = 1782), *2*) children aged 12–17.9 mo (*n* = 1329), *3*) children aged 18–23.9 mo (*n* = 1404), and *4*) children aged 12–23.9 mo (*n* = 2733). For these analyses, treatment arms were pooled as per the study design. We present the impacts by treatment groups in **Supplemental Tables 2–7**.

The sample for the second comparison (2010 with 2014) consisted of 3563 children at baseline (*n* = 2567 for pooled treatment and *n* = 996 for control) and 3560 children at follow-up in 2014 (*n* = 2982 for pooled treatment and *n* = 578 for control) (Figure 1). The analyses were conducted for the full sample and repeated for 3 age groups: *1*) children aged 24–29.9 mo (*n* = 2493), *2*) children aged 30–35.9 mo (*n* = 2129), and *3*) children aged 36–41.9 mo (*n* = 2463).

We assessed the program's effect on the highest attained motor or language milestone using a difference-in-difference (DID) colline fixed-effects model with SEs adjusted for clustering. The DID model compares the change in the outcome over time (2010 with 2012 and 2010 with 2014) between the pooled treatment groups and the control group. The models controlled for household size, house ownership, household head's occupation and education, maternal age and education, and child age and sex.

We also assessed the plausible impact pathways. Variables were selected for inclusion in the pathway analysis based on existing evidence that suggests an association between the selected variables and children's motor or language development and if the program was expected to affect the selected intermediary variables. We used a 2-step approach (14). First, we assessed program impacts on intermediary variables using the same DID colline fixed-effects model. At the household level, the only indicator included was household hunger. Mother-level determinants included maternal underweight, anemia, and stress. Child-level determinants included nutritional status (LAZ/HAZ, WHZ, Hb), having slept under a bednet in the past 24 h, and maternal report of child illness in the past 2 wk (any, diarrhea, and fever). For the younger age group, we also assessed minimum dietary diversity (without CSB consumption), minimum meal frequency, and the consumption of CSB in the past 24 h (these were not collected in the older age group). Age-specific IYCF indicators (e.g., exclusive breastfeeding and initiation of complementary feeding) assessed only for a subset of our sample were not included. Intake of iron-rich food was largely redundant with intake of CSB and excluded. Second, we assessed the association between these determinants and the highest attained motor and language milestones at follow-up using ordinary least squares regression. The pathway analyses were limited to the age subgroups where marginally significant (at least *P* < 0.10) or significant (*P* < 0.05) program effects on child development were found. The 12–17.9 mo and 18–23.9 mo age groups were combined for the analysis of how program impacts were related to motor and language development outcomes to increase power, because the pattern of impacts and relations with the development outcomes were similar in these 2 age groups.

All analyses of program impacts were intent-to-treat. Program impacts were considered statistically significant at *P* < 0.05 and marginally significant at *P* < 0.10. All analyses were conducted in Stata version 14 (StataCorp).

## Results

### Sample characteristics

#### Children 4–23.9 mo of age (in 2010 or 2012)

At baseline, the mean household size was similar between the 2 groups but slightly larger in the treatment group (**Table 1**). Nearly all households were male-headed. A little more than half of household heads had at least some formal education and farming their own land was reported by most as their primary occupation. Mothers of index children were ∼28 y of age and about half had any formal education. The mean age of index children was ∼14 mo and a little less than half were males.

**TABLE 1:** Unadjusted mean key characteristics of households, mothers, and children 4–23.9 mo of age included in the study sample in control and treatment areas^1^

	2010		2012	
	Control (*n* = 795)	Treatment (*n* = 1498)	Control (*n* = 780)	Treatment (*n* = 1496)
Households				
Size, *n*	5.5 ± 2.1	5.6 ± 2.1	5.8 ± 2.0	5.7 ± 2.0
*Tubaramure* participation				
Current beneficiary	—	—	2.5	67.0
Past beneficiary	—	—	1.7	12.6
Never a beneficiary	—	—	95.7	20.2
Own house	97.6	97.1	98.8	96.8
Male head of household	89.4	92.8	92.7	94.1
Household head has had any education	55.9	60.9	58.1	63.7
Household head farms own land	77.5	75.2	78.0	75.2
Mothers				
Age, y	28.4 ± 7.1	28.9 ± 6.9	28.4 ± 6.6	28.7 ± 6.6
Mother has had any education	45.5	49.5	47.3	56.7
Children				
Age, mo	14.4 ± 6.0	14.3 ± 5.8	14.2 ± 5.7	13.6 ± 5.7
Male	47.1	48.0	50.4	52.2

1 All values are percentages or means ± SDs.

At the first follow-up, ∼80% of households in the treatment group reported that they were either participating in *Tubaramure* or had previously participated. Household, maternal, and child characteristics were similar between control and treatment groups at baseline and first follow-up.

#### Children 24–41.9 mo of age (in 2010 or 2014)

At baseline, the mean household size was 5.9 (**Table 2**). More than 90% of households were male-headed and a little more than half of household heads had any formal education. Most household heads had farming their own land as their primary occupation. Mothers of index children were between 30 and 31 y of age and a little less than half had any formal education. The mean age of index children was ∼33 mo and about half were male.

**TABLE 2:** Unadjusted mean key characteristics of households, mothers, and children 24–41.9 mo of age included in the study sample in control and treatment areas^1^

	2010		2014	
	Control (*n* = 996)	Treatment (*n* = 2567)	Control (*n* = 578)	Treatment (*n* = 2982)
Households				
Size, *n*	5.9 ± 2.0	5.9 ± 2.0	6.0 ± 2.0	6.0 ± 2.0
*Tubaramure* participation				
Past beneficiary	—	—	4.2	72.0
Never a beneficiary	—	—	95.8	28.0
Own house	98.2	98.2	96.7	97.7
Male head	92.2	92.0	92.4	92.2
Head has had any education	52.8	57.1	61.0	63.2
Head farms own land	81.9	76.5	73.8	74.8
Mothers				
Age, y	30.5 ± 7.9	31.0 ± 7.8	29.4 ± 6.8	30.3 ± 7.2
Any education	44.1	46.8	48.5	55.0
Children				
Age, mo	32.8 ± 5.6	32.8 ± 5.6	33.4 ± 5.3	33.2 ± 5.4
Male	49.7	47.7	51.0	51.1

1 All values are percentages or means ± SDs.

At second follow-up, 72.0% of households in the treatment group reported that they had previously participated in *Tubaramure*. Household, maternal, and child characteristics were similar between control and treatment groups at baseline and second follow-up.

### Program impact

#### Children 4–23.9 mo of age (in 2010 or 2012)

Among children 4–23.9 mo of age, *Tubaramure* had a significant positive impact on children's language development compared with the control group between 2010 and 2012 (**Table 3**). In age subgroup analyses, the impact of the program on language development was limited to the older children 12–23.9 mo of age (0.7 milestones), with the largest impact among children aged 18–23.9 mo (0.9 milestones). Although there was no program impact on children's motor development for the whole age cohort, age subgroup analyses revealed a marginally significant impact among children 12–23.9 mo of age (0.6 milestones, *P* = 0.08) and a statistically significant impact among those 12–17.9 mo of age (0.9 milestones) (Table 3).

**TABLE 3:** Unadjusted mean motor and language development scores among children 4–23.9 mo of age in 2010 or 2012 living in control and treatment areas and impact estimates^1^

	2010^2^		2012^2^			
	Control	Treatment	Control	Treatment	DID^3^	*P* value
Language milestones						
All (4–23.9 mo)	6.3 ± 4.3 (784)	6.3 ± 4.3 (1493)	6.0 ± 3.5 (779)	6.2 ± 4.1 (1494)	0.4 ± 0.2 (4514)	0.03
4–11.9 mo	2.5 ± 2.2 (301)	2.6 ± 2.2 (553)	3.2 ± 1.9 (306)	2.9 ± 2.2 (625)	−0.2 ± 0.2 (1772)	0.78
12–17.9 mo	7.0 ± 2.9 (196)	6.8 ± 3.1 (463)	6.4 ± 2.7 (234)	6.9 ± 2.8 (451)	0.6 ± 0.3 (1336)	0.03
18–23.9 mo	9.8 ± 3.3 (287)	10.1 ± 3.3 (477)	9.2 ± 2.9 (239)	10.2 ± 3.3 (418)	0.9 ± 0.4 (1406)	0.02
12–23.9 mo	8.7 ± 3.4 (483)	8.5 ± 3.6 (940)	7.8 ± 3.1 (473)	8.5 ± 3.4 (869)	0.7 ± 0.3 (2742)	0.00
Motor milestones						
All (4–23.9 mo)	14.9 ± 6.8 (786)	15.2 ± 6.6 (1483)	14.6 ± 6.0 (766)	14.6 ± 6.5 (1480)	0.0 ± 0.3 (4479)	0.50
4–11.9 mo	8.2 ± 4.8 (304)	8.7 ± 4.6 (552)	9.4 ± 4.6 (303)	8.7 ± 4.6 (623)	−0.6 ± 0.4 (1769)	0.95
12–17.9 mo	17.1 ± 3.9 (196)	17.2 ± 3.7 (457)	16.2 ± 3.6 (231)	17.2 ± 3.6 (445)	0.9 ± 0.4 (1321)	0.02
18–23.9 mo	20.5 ± 3.4 (286)	20.9 ± 3.5 (474)	19.9 ± 3.3 (232)	20.6 ± 3.2 (412)	0.3 ± 0.5 (1389)	0.26
12–23.9 mo	19.1 ± 4.0 (482)	19.1 ± 4.0 (931)	18.1 ± 3.9 (463)	18.8 ± 3.8 (857)	0.6 ± 0.4 (2710)	0.08

1 DID, difference-in-difference.

2 Values are means ± SDs (*n*).

3 Values are coefficients ± SEs and number of observations from DID estimates applying listwise deletion of observations missing information in 2010 or 2012 on any of the covariates included in the model.

#### Children 24–41.9 mo of age (in 2010 or 2014)

For children 24–41.9 mo of age in 2010 or 2014, the program did not have an overall impact on language development. However, in age subgroup analyses there was a positive program impact on language development among children 24–29.9 mo of age (1.0 milestones) (**Table 4**). Among children 24–41.9 mo of age, *Tubaramure* had a marginally significant positive impact on motor development (0.4 milestones, *P* = 0.09) (Table 4). In age subgroup analyses, this marginally significant positive impact was limited to children 24–29.9 mo of age (0.4 milestones, *P* = 0.09).

**TABLE 4:** Unadjusted mean motor and language development scores among children 24–41.9 mo of age in 2010 or 2014 living in control and treatment areas and impact estimates^1^

	2010^2^		2014^2^			
	Control	Treatment	Control	Treatment	DID^3^	*P*
Language milestones						
All (24–41.9 mo)	15.3 ± 3.9 (989)	15.7 ± 4.1 (2256)	16.4 ± 3.8 (571)	17.0 ± 3.6 (2959)	0.3 ± 0.3 (7014)	0.18
24–29.9 mo	13.4 ± 3.5 (367)	13.6 ± 3.8 (956)	14.0 ± 3.8 (168)	15.0 ± 3.8 (992)	1.0 ± 0.3 (2459)	0.00
30–35.9 mo	15.4 ± 3.7 (289)	16.0 ± 3.8 (725)	16.6 ± 3.6 (206)	17.3 ± 3.2 (909)	0.0 ± 0.5 (2118)	0.51
36–41.9 mo	17.3 ± 3.4 (333)	17.8 ± 3.2 (875)	18.3 ± 2.9 (197)	18.7 ± 2.6 (1058)	0.1 ± 0.6 (2437)	0.45
Motor milestones						
All (24–41.9 mo)	24.0 ± 3.5 (989)	24.2 ± 3.3 (2552)	24.5 ± 3.0 (549)	25.0 ± 3.2 (2902)	0.4 ± 0.3 (6934)	0.09
24–29.9 mo	22.9 ± 3.4 (362)	23.0 ± 3.0 (953)	23.6 ± 2.8 (164)	23.9 ± 3.1 (966)	0.4 ± 0.3 (2423)	0.09
30–35.9 mo	23.8 ± 3.5 (290)	24.2 ± 3.3 (725)	24.2 ± 2.9 (199)	25.0 ± 3.2 (888)	0.3 ± 0.5 (2092)	0.25
36–41.9 mo	25.3 ± 3.3 (337)	25.4 ± 3.1 (874)	25.8 ± 2.8 (186)	26.1 ± 2.9 (1048)	0.4 ± 0.4 (2419)	0.17

1 DID, difference-in-difference.

2 Values are mean ± SD (*n*).

3 Values are coefficients ± SEs and number of observations from DID estimates applying listwise deletion of observations missing information in 2010 or 2014 on any of the covariates included in the model.

### Program impact pathways

#### Children 12–23.9 mo of age (in 2010 or 2012)

Among children 12–23.9 mo of age (the age subgroup with positive program impacts on both motor and language development), *Tubaramure* had a significant impact on the household and child outcomes assessed, but not on maternal outcomes. The program resulted in a lower score on the household hunger scale among households in the treatment compared with the control group [0.3 points on the household hunger scale (effect marginally significant)] (**Table 5**). Among children 12–23.9 mo of age, *Tubaramure* resulted in higher WHZ (0.2 SD) and Hb (0.4 g/dL). In this age group, the program also resulted in significantly higher proportions of children who ate CSB [44.2 percentage points (pp)], had a minimally diverse diet (8.2 pp) (effect marginally significant), and met the requirement for minimum meal frequency (13.6 pp). Lastly, the program also resulted in a lower prevalence of any illness (−12.0 pps) or fever (−15.1 pp) among children in the treatment compared with the control group.

**TABLE 5:** Two-stage analysis of impact pathways of the effect of *Tubaramure* on motor and language development among children 12–23.9 mo of age^1^

	2010^2^		2012^2^		Program impact (DID)^3,4^ (*n* = 2751)	Association with attained motor milestone^3,5^ (*n* = 1310)	Association with attained language milestone^3,5^ (*n* = 1332)
	Control (*n* = 488)	Treatment (*n* = 942)	Control (*n* = 474)	Treatment (*n* = 869)			
Household level							
Household hunger scale (0–6)	1.6 ± 1.6	1.4 ± 1.5	1.2 ± 1.6	0.8 ± 1.3	−0.3 ± 0.2*	−0.1 ± 0.1**	−0.1 ± 0.1*
Mother level							
Mother is underweight (BMI < 18.5 kg/m^2^)	14.7	15.7	15.8	18.3	1.6 pp	−0.4 ± 0.3	0.1 ± 0.2
Mother is anemic^6^	37.5	27.8	54.9	41.2	−2.3 pp	−0.2 ± 0.2	−0.2 ± 0.1**
Mother is stressed (Self Reporting Questionnaire >10)	18.1	16.5	12.0	10.7	0.0 pp	−0.0 ± 0.4	0.1 ± 0.3
Child level							
Height-for-age *z* score	−2.5 ± 1.2	−2.4 ± 1.2	−2.6 ± 1.3	−2.5 ± 1.1	0.0 ± 0.1	0.7 ± 0.1***	0.3 ± 0.1***
Weight-for-height *z* score	−0.4 ± 1.2	−0.4 ± 1.1	−0.5 ± 1.2	−0.4 ± 1.0	0.2 ± 0.1**	0.7 ± 0.1***	0.2 ± 0.1***
Hemoglobin, g/dL	10.6 ± 1.4	10.7 ± 1.5	9.8 ± 1.7	10.3 ± 1.6	0.4 ± 0.2***	0.3 ± 0.1***	0.1 ± 0.0***
Minimum dietary diversity	22.4	27.3	30.3	43.8	8.2 pp*	0.6 ± 0.2***	0.8 ± 0.2***
Minimum meal frequency	3.3	3.6	35.4	49.6	13.6 pp***	0.3 ± 0.2*	0.4 ± 0.1***
Consumed CSB in past 24 h	3.4	3.8	5.1	50.1	44.2 pp***	0.6 ± 0.2***	0.8 ± 0.2***
Slept under bednet in past 24 h	33.3	42.5	37.8	45.2	−3.4 pp	0.6 ± 0.2***	0.3 ± 0.2**
Had any illness in past 2 wk	40.9	46.3	54.9	49.3	−12.0 pp***	−0.4 ± 0.1***	−0.2 ± 0.2*
Had a fever in past 2 wk	30.7	32.0	43.5	30.1	−15.1 pp***	−0.5 ± 0.2***	−0.1 ± 0.2
Had diarrhea in past 2 wk	26.6	30.2	29.9	28.9	−5.6 pp	−0.7 ± 0.2***	−0.3 ± 0.2*

1 *,**,***Different from the control group or association with attained milestone: *P < 0.10, **P < 0.05, ***P < 0.01. CSB, corn–soy blend; DID, difference-in-difference; Hb, hemoglobin; pp, percentage point.

2 Values are percentages or means ± SDs. Sample size varies from *n* = 826 to 942 in the treatment arms and from *n* = 408 to 488 in the control arm in 2010. Sample size varies from *n* = 798 to 869 in the treatment arms and from *n* = 431 to 474 in the control arm in 2012.

3 Values are coefficients ± SEs or pp applying listwise deletion of observations with missing information in 2010 or 2012 on any of the covariates included in the model.

4 Sample size varies from *n* = 2445 to 2751.

5 Estimates for children with data from 2012 only. Sample size varies from *n* = 1200 to *n* = 1310 for association with attained motor milestone, and from *n* = 1219 to *n* = 1332 for association with attained language milestone.

6 Maternal anemia was defined as Hb <12 g/dL for nonpregnant women, women who did not know whether they were pregnant, and women with missing information on pregnancy, and as Hb <11 g/dL for pregnant women.

These intermediate outcomes were associated (either significantly or marginally significantly) with children's motor and language development in the expected directions (Table 5). Household hunger was associated with lower motor and language milestones. Related to children's nutritional status, a 1-SD increase in WHZ scores and a 1-g/dL increase in Hb were associated with 0.7 and 0.3 higher attained motor milestones and 0.2 and 0.1 higher attained language milestones, respectively. Along the diet pathway, having consumed CSB, having a minimally diverse diet, and meeting the minimum meal frequency requirement were associated with higher attained motor and language milestones. Along the illness pathway, having had any illness in the previous 2 wk was associated with lower attained motor and language milestones (−0.4 and −0.2, respectively). Fever, specifically, was associated with lower attained motor milestones (−0.5), but not with language milestones.

#### Children 24–29.9 mo of age (in 2010 or 2014)

Among children 24–29.9 mo of age (the age subgroup in which there were positive program impacts on motor and language development) *Tubaramure* resulted in a significantly lower prevalence of maternal underweight (−6.6 pp) and prevalence of any illness, fever, and diarrhea (−18.4 pp, −14.6 pp, and −15.8 pp, respectively) among children and a higher mean HAZ score (0.3 *z* scores). Although the program had a significant positive impact on maternal underweight in this age group, maternal underweight was not associated with children's motor or language development. Children's nutritional status and health were, however, associated with children's developmental outcomes in the expected directions, suggesting plausible program impact pathways (**Table 6**). A 1-unit increase in HAZ was associated with 0.6 and 0.7 higher attained motor and language development scores, respectively. Having had an illness in the previous 2 wk was associated with significantly lower attained motor and language milestones (−0.5 and −0.6, respectively). For language development, having had a fever or having had diarrhea in the previous 2 wk were also significantly associated with lower attained language milestones (−0.4 and −0.9, respectively).

**TABLE 6:** Two-stage analysis of impact pathways of the effect of *Tubaramure* on motor and language development among children 24–29.9 mo of age in 2010 or 2014^1^

	2010^2^		2014^2^			Association with attained motor milestone^3^, ^5^ (*n* = 1124)	Association with attained language milestone^3^, ^5^ (*n* = 1154)
	Control (*n* = 367)	Treatment (*n* = 956)	Control (*n* = 170)	Treatment (*n* = 998)	Program impact^3^, ^4^ (*n* = 2469)		
Household level							
Household hunger scale (0–6)	1.7 ± 1.6	1.3 ± 1.5	1.1 ± 1.6	0.7 ± 1.3	−0.1 ± 0.2	−0.1 ± 0.1	−0.3 ± 0.1***
Mother level							
Mother is underweight, BMI <18.5 kg/m^2^	10.4	14.0	17.9	12.6	−6.6 pp**	−0.1 ± 0.3	−0.4 ± 0.4
Mother is anemic^6^	34.2	28.0	48.4	41.0	−1.0 pp	−0.3 ± 0.1**	−0.2 ± 0.2
Mother is stressed (Self-Reporting Questionnaire >10)	20.6	16.3	22.0	15.2	−1.0 pp	−0.9 ± 0.3***	−0.5 ± 0.3*
Child level							
Height-for-age *z* score	−2.5 ± 1.2	−2.4 ± 1.2	−2.7 ± 1.1	−2.3 ± 1.2	0.3 ± 0.1***	0.6 ± 0.1***	0.7 ± 0.1***
Weight-for-height *z* score	−0.2 ± 1.0	−0.3 ± 1.0	−0.1 ± 1.0	−0.1 ± 1.0	0.1 ± 0.1	0.2 ± 0.1**	0.0 ± 0.1
Hemoglobin, g/dL	10.6 ± 1.6	11.0 ± 1.4	10.4 ± 1.6	10.7 ± 1.5	−0.1 ± 0.2	0.1 ± 0.1**	0.2 ± 0.1***
Slept under bednet in past 24 h	32.7	41.8	72.9	82.6	1.5 pp	0.0 ± 0.2	0.1 ± 0.3
Had any illness in past 2 wk	40.2	41.8	46.5	29.6	−18.4 pp***	−0.5 ± 0.2**	−0.6 ± 0.3**
Had a fever in past 2 wk	31.6	25.8	37.6	18.6	−14.6 pp***	−0.3 ± 0.3	−0.4 ± 0.3*
Had diarrhea in past 2 wk	18.0	20.1	25.3	11.4	−15.8 pp***	−0.3 ± 0.3	−0.9 ± 0.4**

1 *,**,***Different from the control group or association with attained milestone: *P < 0.10, **P < 0.05, ***P < 0.01. DID, difference-in-difference; Hb, hemoglobin; pp, percentage point.

2 Values are percentages or means ± SDs. Sample size varies from *n* = 684 to 956 in the treatment arms and from *n* = 260 to 367 in the control arm in 2010. Sample size varies from *n* = 807 to 998 in the treatment arms and from *n* = 123 to 170 in the control arm in 2014.

3 Values are coefficient ± SE or pp applying listwise deletion of observations with missing information in 2010 or 2012 on any of the covariates included in the model.

4 Sample size varies from *n* = 1859 to 2469.

5 Estimates for children with data from 2014 only. Sample size varies from *n* = 894 to *n* = 1124 for association with attained motor milestone, and from *n* = 924 to *n* = 1154 for association with attained language milestone.

6 Maternal anemia was defined as Hb <12 g/dL for nonpregnant women, women who did not know whether they were pregnant, and women with missing information on pregnancy, and as Hb <11 g/dL for pregnant women.

## Discussion

The multisectoral FA-MCHN program in Burundi significantly improved children's motor and language development although the effects varied by outcome and child age. Effect sizes were generally small (27) but comparable with those found in other studies with children in similar age ranges. In our study, program effects were limited to children in the older age subgroups (12–17.9 mo, 18–23.9 mo, and 24–29.9 mo) as children acquired more language and motor skills. For language development, effect sizes for the 12–17.9 mo and the 18–23.9 mo group were 0.22 and 0.28, respectively and were similar to those seen in evaluations of a BCC intervention in Bangladesh and a combined BCC and multiple micronutrient powder intervention in Pakistan (13, 28). Program impact on motor development was largest among children 12–17.9 mo of age, around the time children attain the motor milestone of independent walking (effect size = 0.27), and was again similar to that found in the aforementioned Bangladesh and Pakistan intervention studies (13, 28). Among older children (24–41.9 mo of age), we found a marginally significant program impact on motor, but not on language, development. Among children 24–29.9 mo of age we found a positive program impact on language development and marginally significant positive program impact on motor development. However, effect sizes were again small (0.27 and 0.12 for language and motor development, respectively). There was no detectable program impact on the development of children >30 mo of age. Advancement of 1 language milestone translated to about a 1.5-mo advancement in language development and 1 motor milestone to about a 1.1-mo advancement in motor development. This translated to advances in development associated with the program of ∼0.5–1.5 mo for language development and 0.5–1.0 mo for motor development across the age groups.

The program impacts detected among children younger than 30 mo of age could translate into later meaningful improvements in functional outcomes later in life. There is some evidence to support that improvements in early child development outcomes predict outcomes in later childhood and adulthood (29–31). A study in Bangladesh demonstrated that motor and language development predicted later cognitive outcomes, although the sizes of the associations were small in the case of motor development (30). In Guatemala the provision of supplementary food in early childhood also resulted in better reading comprehension and reasoning in adulthood and, among men, greater wage-earnings (31). It is possible that the impacts on child development outcomes found in this study among children <30 mo of age could translate into meaningful outcomes in later childhood or into adulthood; however, we would need to follow these children at later ages to determine the longer-term effects of the program on later functional outcomes.

The lack of impact among children older than 30 mo could be due to the sensitivity of the assessment tools or to the timing of the survey in relation to program participation. It is possible that the assessment tools used in this study were more sensitive to detecting program impacts on language and motor development among younger children than among older children in this setting. This was also seen in the Bangladesh study where larger program impacts were seen among younger children (13). In addition, in our study, children in the oldest age group had achieved a mean of 17–19 out of 21 language milestones and between 25 and 26 out of 30 motor milestones, thus this scale may not have been sensitive enough to detect program impact in the older age groups. It is also possible that concurrent or recent program participation is necessary for the program effects on child development outcomes that were seen in this study. The second follow-up included children 24–41.9 mo of age. Program participation ended at 23.9 mo of age and thus children 24–29.9 mo of age had been more recently exposed to the program than those older than 30 mo. In a setting such as Burundi with many risk factors for suboptimal development, continuous efforts may be needed to sustain developmental gains.

Our analyses of program impact pathways included household-, mother-, and child-level determinants. Most of the household-, mother-, and child-level determinants were associated with language and/or motor milestones but these associations varied by age group and developmental outcome. Overall, our findings suggest that *Tubaramure*’s impact on child development operated primarily through improvements in child diet and nutrition and health status.

Our analysis of child-level determinants suggests plausible pathways to improved development through the program's impacts on improving IYCF practices (children 12–23.9 mo of age), reducing morbidity (children 12–23.9 and 24–29.9 mo of age), and improving nutritional status (WHZ and Hb for children 12–23.9 mo of age and HAZ for children 24–29.9 mo of age). The study design does not allow us to establish with certainty that the program operated through these intermediary variables, but the findings suggest plausible pathways. The results are in line with other evidence that suggests that improvements in IYCF practices and nutritional status (e.g., reductions in stunting and anemia) and reductions in morbidity (e.g., diarrhea and malaria) are associated with better child development outcomes (2, 4, 8, 32, 33). For example, a study in Bangladesh that used the same development measures demonstrated that a large-scale BCC program designed to improve IYCF practices improved children's dietary diversity and intake of iron-rich foods and both motor and language development. The results of the path analyses conducted by the authors of that study found that the improvements in children's development were due in part to improvements in children's diet diversity and intake of iron-rich foods (13). In South Africa, the provision of a fortified maize porridge for 6 mo to children 6–12 mo of age increased serum ferritin, Hb, and motor development (12). Lastly, higher incidences of malaria and diarrhea have been negatively associated with development outcomes (2). The incidence of malaria has been associated with poorer cognitive function (34) and with lower motor and language development scores in young children (6). Likewise, a higher incidence of diarrhea has also been negatively associated with development outcomes (2). In our study, having slept under a bednet and not having had a fever were significantly associated with higher motor and language development scores among children 12–23.9 mo of age, but not among those 24–29.9 mo of age (aside from a marginally significant association with language development). Although we did not find a significant program impact on children aged 12–23.9 mo having slept under a bednet, there was a positive program impact on the prevalence of fever in this age group, indicating that the reduction of fever was a plausible program impact pathway through which *Tubaramure* worked to positively affect children's motor and language development. In summary, the significant program impacts and associations with motor and language development suggest plausible program impact pathways through positive program impacts on children's diets, health, and nutritional status. Future programs in contexts similar to Burundi should include interventions specifically designed to improve IYCF practices and reduce anemia, stunting, and morbidity during the first 1000 d.

There is some evidence in the literature of associations between maternal nutritional status or stress and children's development (2, 10). In Bangladesh, supplementary food provided to underweight mothers early, rather than late, in pregnancy led to better development outcomes at 7 mo, although the effects were no longer significant at 18 mo of age (11). Maternal anemia and iron deficiency anemia have been linked to lower levels of responsiveness of caregivers to their infants and, in 1 case, with delayed infant development at 9 mo (35). It has also been suggested that exposure to stress in utero or postnatally can lead to changes in brain development that can negatively affect child development (36–38). In our study, we found that maternal anemia and stress, but not underweight, were associated with lower attained motor and language milestones although the associations varied by child age. Maternal anemia was associated with lower attained language milestones among children 12–23.9 mo of age and with lower attained motor milestones among children 24–29.9 mo of age. Maternal stress was associated with both lower attained motor and language milestones among children 24–29.9 mo of age. Despite significant associations in the expected directions, the program did not significantly affect these determinants in these age groups, suggesting that the program worked through different pathways to improve children's development outcomes. It is possible that larger impacts on child development could be attained if future programs also effectively address these maternal factors in addition to the child-level factors.

Although this study used a rigorous cluster-randomized controlled study design and collected data along the hypothesized impact pathways to assess their plausibility, there are a few study limitations to note. First, the study used repeated cross-sections rather than a longitudinal design and thus the same children were not followed over time. A longitudinal design would have had the advantage of being able to examine the impact pathways over time, such as the relation between children's diets and later development outcomes. In addition, bias due to potential cohort effects would have been reduced had we used a longitudinal design. However, this is unlikely to be a concern because we used a randomized design which included a control group. Thus, any cohort effects would be expected to be similar between the treatment and control groups. Second, we did not collect data on other key potential determinants of child development, including but not limited to factors such as iron deficiency anemia, iodine deficiency, and malaria infection, which were beyond the scope of the study. Lastly, the developmental outcomes used in this study relied on maternal report which could have led to biased reporting due to knowledge of receipt of treatment, social desirability, or common reporter bias. However, because this program did not directly address child development or mention developmental milestones, it is unlikely that this influenced caregiver report of attained motor or language milestones. In addition, the use of a control group limits the potential influence of any bias due to social desirability or common reporter bias on the impact estimates.

The evidence from this study demonstrates that multisectoral FA-MCHN programs can positively affect motor and language development among children <3 y of age. It has been suggested that the best way to reach this age group with early child development interventions is to incorporate them into existing health and nutrition programs (10, 39). Early child development interventions could also be linked to other types of programs such as social safety net or agriculture programs (7, 10, 39–40). It is possible that adding specific interventions to improve child stimulation and other caregiving practices to these types of programs may have additive or synergistic impacts on child development outcomes (10, 39). FA-MCHN programs should consider the inclusion of early child development interventions in addition to the BCC, food, and health interventions provided to maximize the effectiveness on child development in both the short and the long term. Improving children's early development can lead to long-term positive impacts on cognitive skills, schooling, and wage earnings and thus should be prioritized along with improving children's health and nutritional status.

## Supplementary Material

nxz133_Supplemental_File

## Notes

The study was made possible by the generous support of the American people through the support of the Office of Food for Peace, Bureau for Democracy, Conflict, and Humanitarian Assistance, and the Office of Health, Infectious Diseases, and Nutrition, Bureau for Global Health, US Agency for International Development, under terms of Cooperative Agreement No. AID-OAA-A-12-00005, through the Food and Nutrition Technical Assistance III Project, managed by FHI 360. Funding was also received from the CGIAR Research Program on Agriculture for Nutrition and Health led by the International Food Policy Research Institute.

Author disclosures: DKO, JLL, LB, and MTR, no conflicts of interest.

Supplemental Figure 1 and Supplemental Tables 1–7 are available from the “Supplementary data” link in the online posting of the article and from the same link in the online table of contents at https://academic.oup.com/jn/.

Abbreviations used: BCC, behavior change communication; CSB, corn–soy blend; DID, difference-in-difference; FA-MCHN, food-assisted maternal and child health and nutrition; HAZ, height-for-age *z* score; Hb, hemoglobin; IYCF, infant and young child feeding; pp, percentage point; TNFP, *Tubaramure* no food during pregnancy; T18, *Tubaramure* until 18 mo; T24, *Tubaramure* until 24 mo; USAID, US Agency for International Development; WHZ, weight-for-height *z* score.
