# Supplementary material for: A Multisectoral Food-Assisted Maternal and Child Health and Nutrition Program Targeted to Women and Children in the First 1000 Days Increases Attainment of Language and Motor Milestones among Young Burundian Children
Source: J Nutr. 2019 Jul 3;149(10):1833–42. doi: 10.1093/jn/nxz133 (PMC6768810; doi:10.1093/jn/nxz133)

Supplementary data

Supplemental Table 1 Motor and language development scales

#	Motor Milestone	Language Milestone
1	Sit up and hold head straight	Make sounds while playing alone
2	Lift head and chest when lying on the belly	Make sounds like da, ba, ga, ka, ma
3	Turn from the belly toward the back	Make sounds like ma-ma, da-da, ba-ba
4	Keep head straight, without wavering, when held sitting	Imitate simple sounds like da, ba, or ma, or repeat them
5	Move with the belly on the floor (just like swimming)	When holding something in his/her hand, will give the item when requested
6	Sit supported by someone or something	Say one word
7	Sit on his/her own	Say goodbye at the right time waving his/her hand
8	Lift his/her belly when lying on the stomach, and hold on with hands, feet, or knees	Point and make sounds when he/she wants something
9	While lying on the belly, head and chest up, can move using arms and legs	Point to a cat or chicken when asked to do it
10	Crawl on all fours	Say three words
11	Stand leaning on someone or something	Point to a person walking if asked to do it
12	Walk when both hands are held	Say six words
13	Walk when one hand is held	Use pronouns “I” and “you”
14	Stand alone, without help, for a short time	Constantly ask for names of objects
15	Stand alone, without help, for a longer period of time	Start a lot of questions with “What,” “Where,” and “Who”
16	Bend at the waist and stand back up without falling (knees are straight or slightly bent)	Say a lot of words (20 or more)
17	Take a few steps alone, without help from anyone or anything	Use plurals when talking
18	Run	Tell what a knife is used for
19	Climb stairs walking on feet (not crawling)	Say full name (last and first names)
20	Throw a ball (hand lifted to the ear)	Say opposite of word “big”
21	Go up and down the stairs on feet (not crawling)	Talk about things that took place in the past (e.g., day before) using the correct conjugation
22	Kick a ball forward	
23	Walk forward following a straight line (can take 10 steps)	
24	Jump on both feet (both feet up at the same time)	
25	Stand on one foot for several seconds	
26	Walk backwards following a straight line (can take 10 steps)	
27	Walk on tiptoe for four steps (heels raised)	
28	Jump four times without heels touching the ground	
29	Jump on one leg 20 times in a row	
30	Skip using alternate legs	

Supplementary data

Supplemental Table 2 Unadjusted mean motor and language development scores among children 4-23.9 mo of age in 2010 or 2012 living in control and *Tubaramure* 24 (T24) groups and impact estimates

	2010[1]		2012[1]			
	Control	T24	Control	T24	DID[2]	*P*-value
Language milestones						
All (4-23.9 mo)	6.3±4.3 (784)	6.2±4.2 (358)	6.0±3.5 (779)	6.1±4.1 (379)	0.6±0.3	0.01
4-11.9 mo	2.5±2.2 (301)	2.5±2.1 (134)	3.2±1.9 (306)	3.0±2.1 (165)	-0.1±0.3	0.65
12-17.9 mo	7.0±2.9 (196)	6.9±3.2 (104)	6.4±2.7 (234)	6.8±2.8 (114)	0.4±0.5	0.20
18-23.9 mo	9.8±3.3 (287)	9.6±3.4 (120)	9.2±2.9 (239)	10.5±3.3 (100)	1.6±0.6	0.01
12-23.9 mo	8.7±3.4 (483)	8.3±3.6 (224)	7.8±3.1 (473)	8.5±3.5 (214)	1.0±0.4	0.01
Motor milestones						
All (4-23.9 mo)	14.9±6.8 (786)	15.2±6.6 (355)	14.6±6.0 (766)	14.4±6.3 (377)	0.1±0.4	0.45
4-11.9 mo	8.2±4.8 (304)	8.6±4.4 (134)	9.4±4.6 (303)	8.9±4.3 (165)	-0.7±0.4	0.95
12-17.9 mo	17.1±3.9 (196)	17.2±3.5 (103)	16.2±3.6 (231)	16.9±3.7 (113)	0.9±0.7	0.09
18-23.9 mo	20.5±3.4 (286)	20.9±3.8 (118)	19.9±3.3 (232)	20.6±3.1 (99)	0.3±0.7	0.31
12-23.9 mo	19.1±4.0 (482)	19.2±4.1 (221)	18.1±3.9 (463)	18.7±3.9 (212)	0.5±0.6	0.19

[1] Values are mean ± SD (*n*). [2] Values are coefficient ± SE from DID estimates applying listwise deletion of observations missing information in 2010 or 2012 on any of the covariates included in the model. Covariates included household size, house ownership, household head's occupation and education, maternal age and education, and child age and sex. DID, difference-in-difference.

Supplementary data

Supplemental Table 3 Unadjusted mean motor and language development scores among children 4-23.9 mo of age in 2010 or 2012 living in control and *Tubaramure* 18 (T18) groups and impact estimates

	2010[1]		2012[1]			
	Control	T18	Control	T18	DID[2]	*P*-value
Language milestones						
All (4-23.9 mo)	6.3±4.3 (784)	6.4±4.3 (761)	6.0±3.5 (779)	6.3±4.1 (748)	0.4±0.2	0.04
4-11.9 mo	2.5±2.2 (301)	2.6±2.1 (274)	3.2±1.9 (306)	2.8±2.0 (302)	-0.3±0.3	0.84
12-17.9 mo	7.0±2.9 (196)	6.8±3.1 (236)	6.4±2.7 (234)	7.0±2.6 (217)	0.7±0.3	0.01
18-23.9 mo	9.8±3.3 (287)	10.1±3.3 (251)	9.2±2.9 (239)	10.1±3.4 (229)	0.9±0.4	0.02
12-23.9 mo	8.7±3.4 (483)	8.5±3.6 (487)	7.8±3.1 (473)	8.6±3.4 (446)	0.8±0.3	0.01
Motor milestones						
All (4-23.9 mo)	14.9±6.8 (786)	15.4±6.5 (755)	14.6±6.0 (766)	14.6±6.5 (737)	-0.2±0.3	0.68
4-11.9 mo	8.2±4.8 (304)	8.8±4.5 (274)	9.4±4.6 (303)	8.6±4.6 (300)	-0.6±0.4	0.94
12-17.9 mo	17.1±3.9 (196)	17.0±3.7 (232)	16.2±3.6 (231)	16.9±3.6 (214)	0.8±0.5	0.05
18-23.9 mo	20.5±3.4 (286)	21.1±3.4 (249)	19.9±3.3 (232)	20.5±3.3 (223)	0.1±0.6	0.46
12-23.9 mo	19.1±4.0 (482)	19.2±4.1 (481)	18.1±3.9 (463)	18.7±3.9 (437)	0.4±0.5	0.17

[1] Values are mean ± SD (*n*). [2] Values are coefficient ± SE from DID estimates applying listwise deletion of observations missing information in 2010 or 2012 on any of the covariates included in the model. Covariates included household size, house ownership, household head's occupation and education, maternal age and education, and child age and sex. DID, difference-in-difference

Supplementary data

Supplemental Table 4 Unadjusted mean motor and language development scores among children 4-23.9 mo of age in 2010 or 2012 living in control and *Tubaramure* No Food during pregnancy (TNFP) groups and impact estimates

	2010[1]		2012[1]			
	Control	TNFP	Control	TNFP	DID[2]	*P*-value
Language milestones						
All (4-23.9 mo)	6.3±4.3 (784)	6.3±4.3 (374)	6.0±3.5 (779)	6.0±4.0 (367)	0.2±0.3	0.22
4-11.9 mo	2.5±2.2 (301)	2.7±2.3 (145)	3.2±1.9 (306)	3.1±2.6 (158)	-0.1±0.4	0.63
12-17.9 mo	7.0±2.9 (196)	6.8±3.1 (123)	6.4±2.7 (234)	7.0±3.1 (120)	0.5±0.6	0.17
18-23.9 mo	9.8±3.3 (287)	10.6±3.3 (106)	9.2±2.9 (239)	10.1±3.1 (89)	0.0±0.5	0.54
12-23.9 mo	8.7±3.4 (483)	8.5±3.7 (229)	7.8±3.1 (473)	8.3±3.4 (209)	0.4±0.3	0.14
Motor milestones						
All (4-23.9 mo)	14.9±6.8 (786)	15.0±6.6 (373)	14.6±6.0 (766)	14.7±6.6 (366)	0.3±0.4	0.24
4-11.9 mo	8.2±4.8 (304)	8.8±5.0 (144)	9.4±4.6 (303)	8.9±4.6 (158)	-0.5±0.4	0.91
12-17.9 mo	17.1±3.9 (196)	17.5±3.8 (122)	16.2±3.6 (231)	17.8±3.6 (118)	1.0±0.5	0.03
18-23.9 mo	20.5±3.4 (286)	20.5±3.5 (107)	19.9±3.3 (232)	21.0±3.2 (90)	1.0±0.8	0.09
12-23.9 mo	19.1±4.0 (482)	18.9±4.0 (229)	18.1±3.9 (463)	19.1±3.8 (208)	1.1±0.5	0.02

[1] Values are mean ± SD (*n*). [2] Values are coefficient ± SE from DID estimates applying listwise deletion of observations missing information in 2010 or 2012 on any of the covariates included in the model. Covariates included household size, house ownership, household head's occupation and education, maternal age and education, and child age and sex. DID, difference-in-difference

Supplementary data

Supplemental Table 5 Unadjusted mean motor and language development scores among children 24-41.9 mo of age in 2010 or 2014 living in control and *Tubaramure* 24 (T24) groups and impact estimates

	2010		2014			
	Control	T24	Control	T24	DID[2]	*P*-value
Language milestones						
All (24-41.9 mo)	15.3±3.9 (989)	15.9±4.1 (981)	16.4±3.8 (571)	17.3±3.5 (979)	0.4±0.4	0.15
24-29.9 mo	13.4±3.5 (367)	13.8±4.0 (387)	14.0±3.8 (168)	15.4±3.8 (339)	1.0±0.3	0.00
30-35.9 mo	15.4±3.7 (289)	16.3±3.7 (264)	16.6±3.6 (206)	17.7±3.1 (296)	0.0±0.5	0.48
36-41.9 mo	17.3±3.4 (333)	18.0±3.3 (330)	18.3±2.9 (197)	19.0±2.3 (344)	0.2±0.7	0.36
Motor milestones						
All (24-41.9 mo)	24.0±3.5 (989)	24.1±3.3 (982)	24.5±3.0 (549)	25.1±3.1 (962)	0.5±0.3	0.06
24-29.9 mo	22.9±3.4 (362)	22.9±2.9 (386)	23.6±2.8 (164)	24.1±3.0 (331)	0.6±0.3	0.03
30-35.9 mo	23.8±3.5 (290)	24.2±3.3 (264)	24.2±2.9 (199)	25.1±3.1 (290)	0.4±0.5	0.23
36-41.9 mo	25.3±3.3 (337)	25.3±3.3 (332)	25.8±2.8 (186)	26.1±2.8 (341)	0.5±0.5	0.13

[1] Values are mean ± SD (*n*). [2] Values are coefficient ± SE from DID estimates applying listwise deletion of observations missing information in 2010 or 2014 on any of the covariates included in the model. Covariates included household size, house ownership, household head's occupation and education, maternal age and education, and child age and sex. DID, difference-in-difference

Supplementary data

Supplemental Table 6 Unadjusted mean motor and language development scores among children 24-41.9 mo of age in 2010 or 2014 living in control and *Tubaramure* 18 (T18) groups and impact estimates

	2010		2014			
	Control	T18	Control	T18	DID[2]	*P*-value
Language milestones						
All (24-41.9 mo)	15.3±3.9 (989)	15.2±4.1 (583)	16.4±3.8 (571)	16.9±3.6 (990)	0.6±0.4	0.08
24-29.9 mo	13.4±3.5 (367)	13.0±3.8 (216)	14.0±3.8 (168)	14.9±3.8 (320)	1.5±0.5	0.00
30-35.9 mo	15.4±3.7 (289)	15.2±3.9 (182)	16.6±3.6 (206)	17.2±3.3 (311)	0.6±0.6	0.16
36-41.9 mo	17.3±3.4 (333)	17.8±3.1 (185)	18.3±2.9 (197)	18.5±2.6 (359)	-0.2±0.6	0.60
Motor milestones						
All (24-41.9 mo)	24.0±3.5 (989)	24.0±3.5 (579)	24.5±3.0 (549)	25.0±3.2 (964)	0.5±0.4	0.11
24-29.9 mo	22.9±3.4 (362)	22.7±3.3 (214)	23.6±2.8 (164)	23.8±3.2 (310)	0.7±0.4	0.05
30-35.9 mo	23.8±3.5 (290)	24.1±3.5 (181)	24.2±2.9 (199)	25.1±3.3 (302)	0.6±0.6	0.13
36-41.9 mo	25.3±3.3 (337)	25.4±3.0 (184)	25.8±2.8 (186)	25.9±3.0 (352)	0.1±0.6	0.41

[1] Values are mean ± SD (*n*). [2] Values are coefficient ± SE from DID estimates applying listwise deletion of observations missing information in 2010 or 2014 on any of the covariates included in the model. Covariates included household size, house ownership, household head's occupation and education, maternal age and education, and child age and sex. DID, difference-in-difference

Supplementary data

Supplemental Table 7 Unadjusted mean motor and language development scores among children 24-41.9 mo of age in 2010 or 2014 living in control and *Tubaramure* No Food during Pregnancy (TNFP) groups and impact estimates

	2010		2014			
	Control	TNFP	Control	TNFP	DID[2]	*P*-value
Language milestones						
All (24-41.9 mo)	15.3±3.9 (989)	15.8±3.9 (992)	16.4±3.8 (571)	16.8±3.6 (990)	0.0±0.4	0.47
24-29.9 mo	13.4±3.5 (367)	13.6±3.7 (353)	14.0±3.8 (168)	14.7±3.7 (333)	0.5±0.4	0.09
30-35.9 mo	15.4±3.7 (289)	16.2±3.8 (279)	16.6±3.6 (206)	17.1±3.2 (302)	-0.5±0.6	0.82
36-41.9 mo	17.3±3.4 (333)	17.6±3.2 (360)	18.3±2.9 (197)	18.5±2.8 (355)	0.1±0.7	0.46
Motor milestones						
All (24-41.9 mo)	24.0±3.5 (989)	24.3±3.2 (991)	24.5±3.0 (549)	25.0±3.2 (976)	0.2±0.4	0.32
24-29.9 mo	22.9±3.4 (362)	23.2±3.0 (353)	23.6±2.8 (164)	23.8±3.1 (325)	0.0±0.4	0.52
30-35.9 mo	23.8±3.5 (290)	24.3±3.0 (280)	24.2±2.9 (199)	24.9±3.1 (296)	0.0±0.6	0.48
36-41.9 mo	25.3±3.3 (337)	25.5±3.0 (358)	25.8±2.8 (186)	26.2±2.9 (355)	0.4±0.5	0.22

[1] Values are mean ± SD (*n*). [2] Values are coefficient ± SE from DID estimates applying listwise deletion of observations missing information in 2010 or 2014 on any of the covariates included in the model. Covariates included household size, house ownership, household head's occupation and education, maternal age and education, and child age and sex. DID, difference-in-difference

Supplementary data

Supplemental Figure 1 Study design

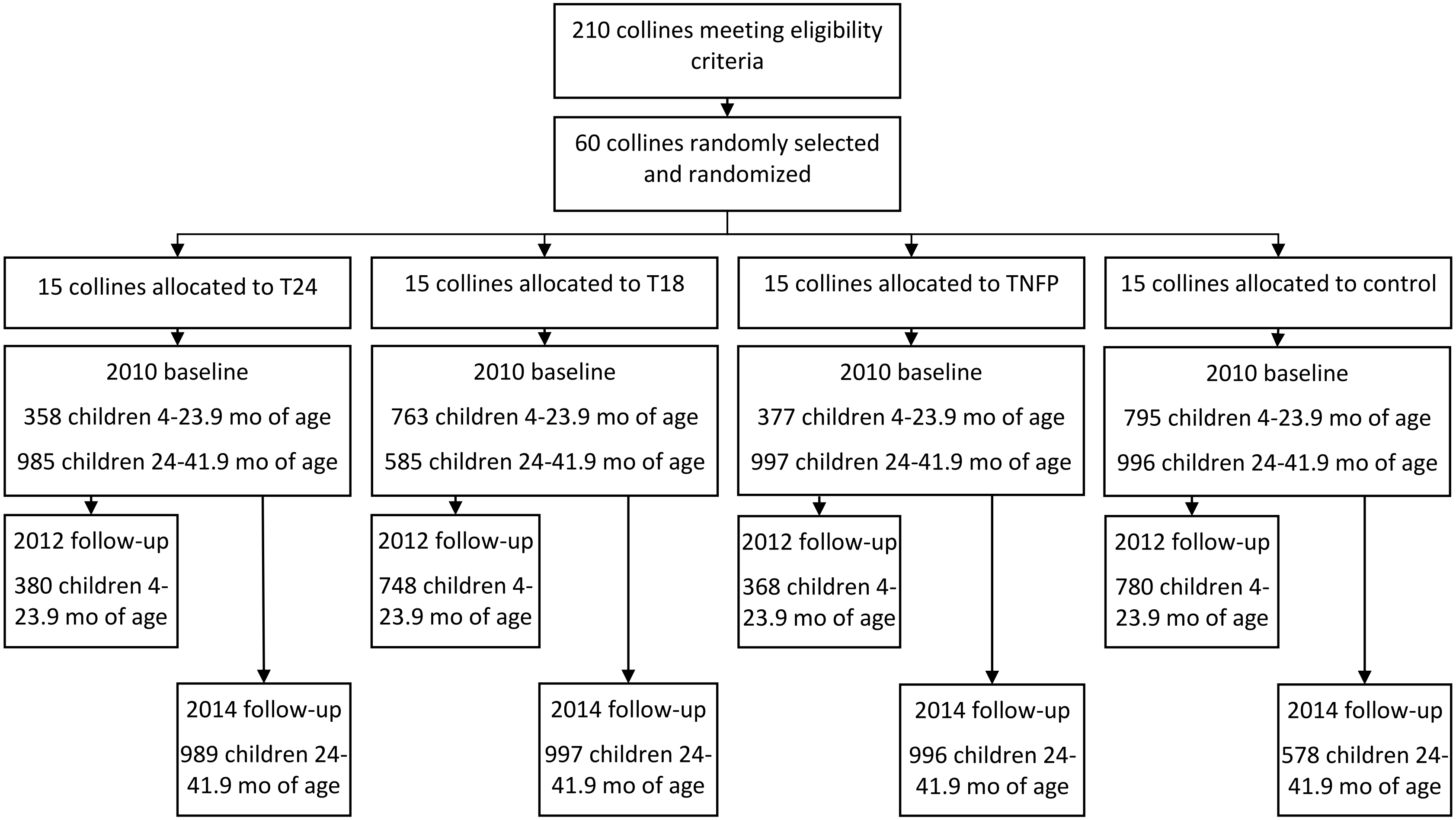

Supplement: nxz133_Supplemental_File [file nxz133_supplemental_file.pdf]
